# Use of childhood adversity and mental health admission patterns to predict suicide in young people

**DOI:** 10.1192/bjo.2025.787

**Published:** 2025-06-20

**Authors:** Anna Tarasenko, Dennis Ougrin

**Affiliations:** Mindly Ltd, London, UK; Youth Resilience Unit, Wolfson Institute of Population Health, Queen Mary University of London, London, UK

**Keywords:** Young person suicide, mental health admission patterns, risk prediction, clinical decision support systems

## Abstract

Dougall et al found that mental health admissions are a strong predictor of suicide risk in young people. The findings can improve machine learning models for predicting suicide risk. Limitations of machine learning models include recent changes in healthcare use patterns during the COVID-19 pandemic and poor long-term predictive value.


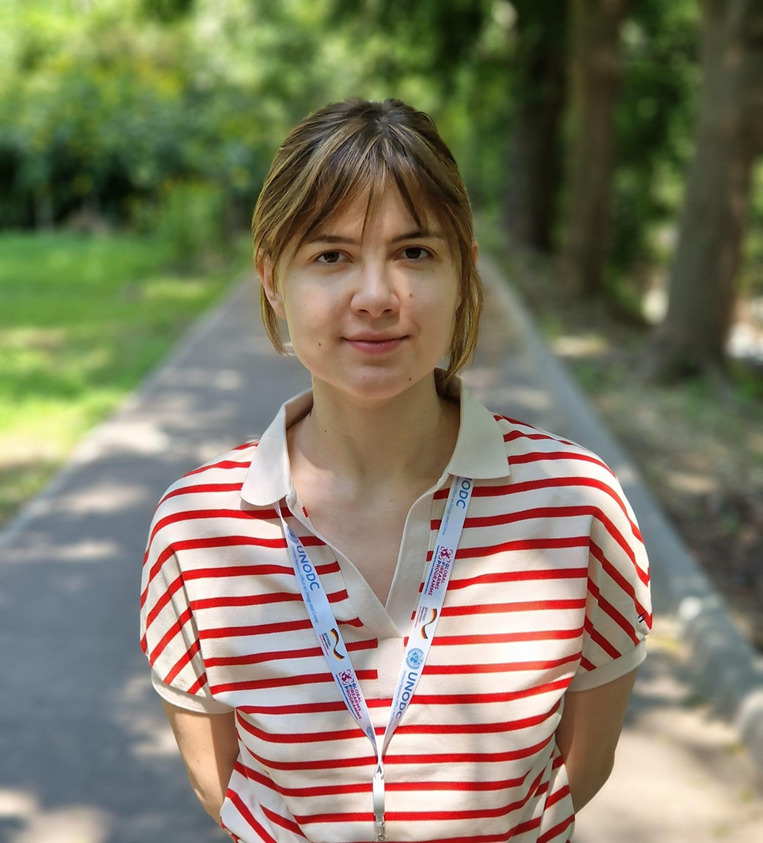


Dougall et al^1^ describe childhood adversity and mental health admission patterns before suicide in young people. Dougall et al conducted a population-based, longitudinal study in Scotland, in which they compared general in-patient and psychiatric records of young people who died by suicide between the years of 1991 and 2017 (cases) and matched controls.^1^ All individuals had to be born after or in 1981. Adverse events were identified in hospital admissions based on the ICD^2^. Adverse events were operationalised in two ways: (a) maltreatment or violence-related codes (MVR) for admissions before 18 years of age or (b) codes suggestive of maltreatment for ages <10 years. Previous studies have strongly supported the notion that childhood adversity can lead to poor mental health outcomes. However, these were predominantly cross-sectional, relied on recall and were heterogeneous.

Research has shown that, in principle, it is possible to use patient electronic health records to design a model to enhance our ability to predict suicide risk. However, this research has been focused on the adult population. Few studies have attempted to do it for children, adolescents and young adults.

## How were the admission patterns different between cases and controls?

In all investigated age groups, cases had more in-patient admissions for adversities and mental health disorders compared with controls. The strongest association for both genders was between mental health admissions and later suicide-related outcomes. Moreover, the odds of suicide-related outcomes increased with the increased number of mental health admissions.

## What was the relationship between mental health and childhood adversity admissions and later outcomes?

The dose–response relationship between suicide and mental health admissions was confirmed, with more mental health admissions increasing the odds of later suicide. When looking at the impact of the type of admissions, it appeared that mental health admissions in men experiencing adversity could act as a protective factor for later suicide, which goes against previously reported data.^3^

Each adversity type multiplied the odds of suicide by 1.9 in men and 2.65 in women. In men, mental health admissions were followed by two or more admissions for MVR admissions, maternal bereavement, accidental poisoning and one MVR admission. In women, mental health admissions were followed by admissions for maternal bereavement, admissions indicating care experience or no fixed abode, and one MVR admission. An interesting finding emerged when looking at people with both mental health and adversity-related admissions. For women, the adversity admissions increased the odds of suicide more than the mental health admissions. In men, this trend was reversed.

## Discussion

Dougall et al’s^1^ paper was founded on a large body of previous research that did not focus specifically on young people. The findings also builds on previous findings that maltreatment is one of the strongest correlates of suicide in children.^4^ Results are in line with other studies of healthcare utilisation that show an increased use of primary care, emergency department and secondary care services before suicide,^5^ with females having more contacts with healthcare services than males.

The mental health category of admissions used by Dougall et al has a broad definition that includes self-harm, which in turn is a strong predictor of later suicide. Because of its heterogeneity, this category warrants further investigation, since it includes a broad range of behaviours likely to have different effects on the risk of suicide.

### Implications for clinical practice

There is a relatively good understanding of many risk and protective factors associated with the risk of suicide-related outcomes. Long in-patient admissions are associated with an increased risk of self-harm in young people.^6^ The COVID-19 pandemic has led to significant changes in help-seeking behaviour for self-harm.^7^ Conversely, we now know what common elements are shared by psychological therapies that reduce self-harm,^8^ and that ensuring continuity of care and offering psychological therapies improves treatment engagement.^9^ The relationship between risk and protective factors and their relative importance within individual patients remains unresolved. This means that Dougall et al’s findings have limited direct implications for clinical practice. However, these findings are an essential stepping stone for creating computerised systems that can supplement decision-making in the clinical setting.

Introducing clinical decision support systems (CDSS) is not new and has been proposed since 1970.^10^ The improvement in the quality of patient records, and increase in the widespread use of machine learning approaches capable of analysing them, mean that CDSS can become a real possibility even in the complex fields of mental health diagnosis and prevention.

Machine learning models differ depending on the type, properties and amount of data they aim to analyse, and the outputs they can produce. Clinicians may be interested not only in the number representing the risk of suicide, which would allow them to create a prioritisation system, but also in understanding the importance of different predictors that were included in the model. Yet, there is a lack of consensus regarding the usability and implementation of prediction models in clinical practice. This may stem from the lack of homogeneity within the research protocols in this area, different machine learning methods used, quality and quantity of the data fed into the models and, most importantly, time scales over which predictions are considered. There is strong evidence to suggest that the performance of machine learning models predicting the risk of suicide depends on the time frame used. These models might be more accurate in a short period of time and much less accurate in longer timeframes.^11^ For studies like that by Dougall et al, the prediction timeframe should be explored further. The strength of effects of different types of admissions might differ at different time points, e.g. straight after the admission versus years later.

The starting point is crucial when creating an machine learning model for risk prediction. Often, models start with a list of possible predictors that affect a particular risk, so creating a comprehensive list of predictors in part defines its performance. The task of the model is to figure out how important each of these predictors is, and at what point. So far, the limited number of suicide risk prediction models relied mainly on demographics, diagnoses, laboratory tests and medication use, although maltreatment appears to be an important factor in machine learning models of suicide-related outcomes.^12^ Studies like the one by Dougall et al are critical because they demonstrate the value of using admission data, among other predictors, when examining the risks of suicide outcomes. Moreover, they highlight the importance of the order of different types of admissions, whereby they looked at different admission combinations in a lifespan and how each of these affects suicide outcomes. They considered mental health admissions only, adverse event admissions only, mental health admission first, adverse event admission second and *vice versa*.

### Limitations

The pattern of admissions changed significantly during the COVID-19 pandemic. At the beginning of the pandemic, there was a sharp decrease in psychiatric hospital admissions and emergency presentations in the UK and worldwide. However, the rate of those presenting with self-harm and suicidal ideation has increased since the pandemic.^7^ Models built on longitudinal data need to account for these changes. Healthcare services are likely to be affected by the patient backlog that accumulated through the pandemic and the consequences of long-COVID.

In conclusion, patterns of admission constitute an important factor that can potentially be used when predicting suicide risk for children and young people. These data can be difficult to interpret, especially when they have high degrees of recall bias. Moreover, the horizon of models built on these data remains limited to shorter prediction timeframes.
